# Low uptake of continuous subcutaneous insulin infusion therapy in people with type 1 diabetes in Ireland: a retrospective cross-sectional study

**DOI:** 10.1186/s12902-020-00573-w

**Published:** 2020-06-23

**Authors:** Katarzyna Anna Gajewska, Kathleen Bennett, Regien Biesma, Seamus Sreenan

**Affiliations:** 1 Division of Population Health Scineces, RCSI: University of Medicine and Health Sciences, Dublin, Ireland; 2 Department of Health Sciences, Global Health, Univeristy Medical Centre Groningen, Groningen, the Netherlands; 3 3U Diabetes, RCSI: University of Medicine and Health Scineces, Dublin, Ireland; 4 Diabetes and Endocrinology. RCSI: University of Medicine and Health Sciences, Connolly Hospital, Dublin, Ireland

**Keywords:** Diabetes mellitus, type 1, Insulin infusion systems, Insulin pump, Epidemiology, Health services research, Cross-sectional studies

## Abstract

**Background:**

The uptake of continuous subcutaneous insulin infusion (CSII) therapy in those with type 1 diabetes varies internationally and is mainly determined by the national healthcare reimbursement systems. The aim of this study is to estimate national and regional uptake of CSII therapy in children, adolescents and adults with type 1 diabetes in Ireland.

**Methods:**

A retrospective cross-sectional study was conducted utilizing the national pharmacy claims database in 2016. Individuals using CSII were identified by dispensing of infusion sets. The uptake of CSII was calculated as the percentage of people with type 1 diabetes claiming CSII sets in 2016, both in children and adolescent (age < 18 years) and adult populations (≥ 18 years). Descriptive statistics including percentages with 95% confidence intervals (CIs) are presented, stratified by age-groups and geographical regions, and chi-square tests used for comparisons.

**Results:**

Of 20,081 people with type 1 diabetes, 2111 (10.5, 95% CI: 10.1–10.9%) were using CSII in 2016. Uptake was five-fold higher in children and adolescents at 34.7% (95% CI: 32.9–36.5%) than in adults at 6.8% (95% CI: 6.4–7.2%). Significant geographical heterogeneity in CSII uptake was found, from 12.6 to 53.7% in children and adolescents (*p <* 0.001), and 2 to 9.6% in adults (*p <* 0.001).

**Conclusions:**

Uptake of CSII in people with type 1 diabetes is low in Ireland, particularly in those ≥18 years. Identification of barriers to uptake, particularly in this group, is required.

## Background

Continuous Subcutaneous Insulin Infusion (CSII) was introduced as a mode of insulin administration for type 1 diabetes in the late 1970s [1]. The International Society of Paediatric and Adolescent Diabetes (ISPAD) recommends CSII as a preferred method of insulin administration for preschool children [2], due to its advantages: better precision and accuracy; flexibility; ease of insulin delivery [1]. Consensus statements from the major international diabetes associations [3] and systematic reviews and meta-analyses [4] suggest a lower risk of hypoglycaemia (including severe events), as well as modest reductions in glycated haemoglobin (HbA_1c_) when comparing CSII to insulin injections in those under 18 years of age [3, 4].

The effectiveness of CSII in adult populations with type 1 diabetes is less evident: systematic reviews and randomized controlled trials (RCTs) of CSII effectiveness in adults with type 1 diabetes reported advantages over multiple daily injections (MDI) such as better control of glucose levels, less hypoglycaemia and quality of life gains [5–7]. Some studies, however, fail to show significant clinical benefit of CSII over MDI, when both randomized groups received the same structured education program [8]. CSII is more complex, requires provision of additional training and education [9], and is a more expensive mode of therapy. A longitudinal analysis of the Swedish National Diabetes Register found the average costs were approximately $4000 more per annum compared to MDI only [10]. However, some studies have suggested that CSII is associated with reduced risk of diabetes-related complications [11, 12], including lower cardiovascular mortality [13].

Notwithstanding the benefits, there is still limited uptake of CSII in those with type 1 diabetes, although there have been increases, mainly in well-developed countries, in those where CSII is reimbursed, and in younger aged populations [1, 14]. From what is already known, the uptake of CSII is usually lower in adults than in children with type 1 diabetes (e.g. in Scotland [15] or Italy [16]), due, in part, to factors such as the health system structure and different reimbursement strategies for children and adolescents compared to adults. In Germany and Austria, the percentage of people using CSII increased from 1% in 1995 to 53% in 2017 across all ages, with the highest uptake in the youngest age group (92% of German and Austrian pre-schoolers) [14]. Comparison between three large paediatric diabetes registries in 2015 showed that 47% of children in the United States (US - T1D Exchange), 41% in Germany and Austria (DPV), and 14% in the English/Welsh National Pediatric Diabetes Audit [17] were using CSII. Evidence from 2017 using data from large diabetes registries found the level of uptake of CSII in adults varied from 9.4% in Scotland [15] to 37% in Germany [14]. As CSII is fully reimbursed in Ireland, as in most of the countries mentioned above (excluding the US), in this study we aimed to estimate the national and regional uptake of CSII in children, adolescents and adults with type 1 diabetes in Ireland.

## Methods

A retrospective cross-sectional study was conducted utilizing the Irish Health Service Executive Primary Care Reimbursement Service (HSE-PCRS) national pharmacy claims database for 2011–2016. The STrengthening the Reporting of OBservational Studies in Epidemiology (STROBE) statement checklist was followed to ensure the quality of conduct and reporting of the study.

### Settings / data sources

The HSE-PCRS pharmacy claims database records (anonymously) monthly dispensed medications from the government funded medication reimbursement schemes in Ireland: Drug Payment (DP), General Medical Services (GMS), and Long-Term Illness (LTI) schemes [18]. People with diabetes have their treatment-related costs fully covered by the state (including insulin, glucometer test strips, insulin pump infusion sets, etc.) mainly through the LTI scheme [19]. LTI and GMS schemes are available to all Irish citizens with diabetes, irrespective of whether their care is provided through the public or private system or in primary or secondary care. The database records basic demographic information, including gender, age group and locality of residence, and the type of medication for diabetes, according to the World Health Organization (WHO) Anatomical Therapeutic Classification (ATC) codes for diabetes (A10) [20]. More detailed information about the HSE-PCRS database can be found elsewhere [18]. Ethical approval to conduct the study was not required as the data were anonymised and permission was provided from the data controllers (HSE-PCRS) for use of the data for this specific study.

### Study population and definitions

The study population included people with type 1 diabetes living and claiming their diabetes-related prescriptions, including CSII sets, in Ireland in 2016. The final population of those with type 1 diabetes (20,081) was estimated as part of another study using similar methodology and the HSE-PCRS data-set. The criteria and definition of a person with type 1 diabetes were complex and included: continuous use of insulin (any type) and glucose test strips (any type); those who used oral hypoglycaemic agents for the 12 months prior to commencing insulin, and those on long-acting insulin only, were excluded from analysis. More details about the methodology, its strengths and limitations, the criteria and definitions are fully described elsewhere [19].

### Statistical analysis

The uptake of CSII was calculated as the percentage of people with type 1 diabetes claiming CSII sets in 2016. Descriptive statistics, including percentages with 95% confidence intervals (CIs), are presented. Type 1 diabetes is most often described in the paediatric population, and there are fundamental differences in the approach, treatments, needs, problems and delivery of diabetes care between children and adults with type 1 diabetes [21]. Thus we decided to stratify the data for the paediatric population (children and adolescents aged under 18 years), and adults (aged 18 and over) separately. In addition, in a previous study on the prevalence of type 1 diabetes in Ireland the same stratification by paediatric and adult cases was used, as well as the stratification by age-groups used by the Irish Central Statistics Office [22] (0–14, 15–24, 25–34, 35–44, 45–54, 55–64, 65–74, 75+) that allowed the prevalence rates for the Irish population to be estimated [19].

For the purposes of this study, analysis was conducted in 18 geographical areas related to 32 Local Health Offices areas [23] (Additional file 1 Table S1). Chi-square tests (including linear test for trend for age) were used to compare uptake of CSII across age groups and geographical regions. Significance at *p <* 0.05 was assumed. The uptake by geographical regions of residence was mapped using ArcGIS software version 10.2.2. SAS statistical software version 9.4 and Microsoft Excel for Mac 2011 were used for analysis.

## Results

There were 2111 people with diabetes claiming CSII sets in 2016, providing an overall uptake of CSII of 10.5% (95% CI: 10.1 to 10.9%; Table 1). The uptake was five-fold higher in those aged < 18 years (34.7, 95% CI: 32.9 to 36.5%) than in those aged ≥ 18 years (6.8, 95% CI: 6.4–7.2%). No information was available on age for *n =* 50 (2%). Of all those using CSII in Ireland, 43% were children and adolescents.

**Table 1:** Uptake (n and %) of Continuous Subcutaneous Insulin Infusion (CSII) therapy in people with type 1 diabetes in Ireland in 2016

Age Group (Years)	CSII therapy users (Total numbers)	People with type 1 diabetes (total numbers)	% Uptake of CSII	95% CI
0–14	702	1846	38.0%	35.8 to 40.2%
15–24	417	2182	19.1%	17.5 to 20.8%
25–34	239	2363	10.1%	8.9 to 11.3%
35–44	277	2831	9.8%	8.7 to 10.9%
45–54	235	2897	8.1%	7.1 to 9.1%
55–64	121	2889	4.2%	3.5 to 4.9%
65–74	55	2688	2.1%	1.5 to 2.6%
75+	15	1948	0.8%	0.4 to 1.2%
TOTAL	**2111**^**a**^	**20,081**^**b**^	**10.5%**	**10.1 to 10.9%**
< 18 years	**899**	**2591**	**34.7%**	**32.9 to 36.5%**
≥18 years	**1162**	**17,053**	**6.8%**	**6.4 to 7.2%**

^a^There were 50 missing data for age (2%) in the uptake of insulin pumps

^b^There were 437 missing data for age (2%) in the prevalence of type 1 diabetes

**Fig. 1:**
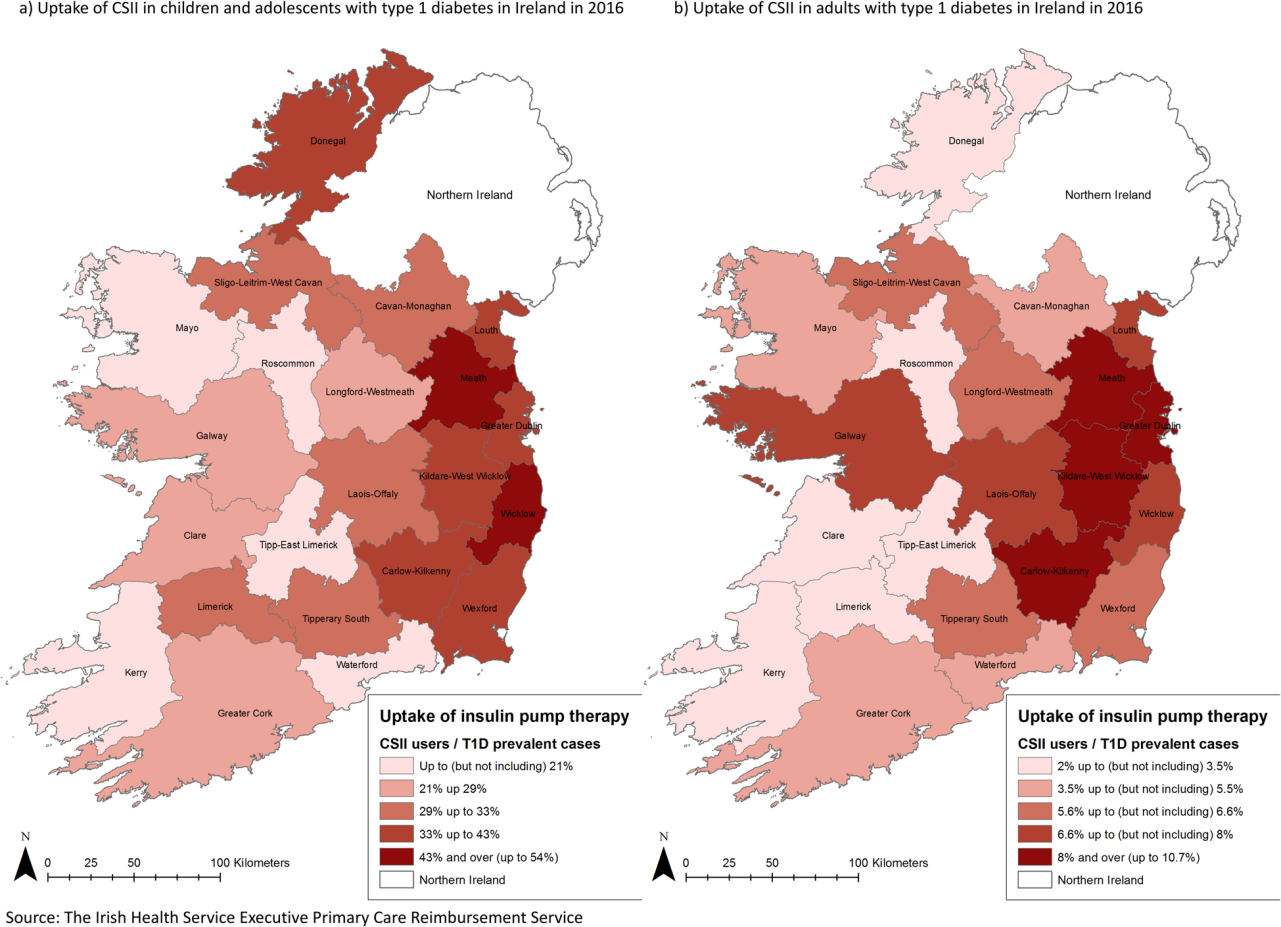
The maps of the uptake of Continuous Subcutaneous Insulin Infusion in children and adolescents and adults with type 1 diabetes in Ireland in 2016. Copyright: Katarzyna Anna Gajewska

The uptake of CSII varied significantly between different age groups (Table 1) and geographical regions (Fig. 1, Table 2). There was a significant linear association of decreasing CSII use with increasing age (*χ*^2^ = 1678.7, *p <* 0.001). The highest uptake was in the youngest population (38% in children aged ≤ 14 years), through to 2.1 and 0.8% in the oldest age groups (people aged 65–74 and over 75 years respectively; Table 1).

**Table 2:** Geographical differences in the uptake of continuous subcutaneous insulin infusion in a whole population, children and adolescents, and adults over 18 with type 1 diabetes in Ireland in 2016

Geographical area	Uptake of CSII – All (%); (95% CI)	Uptake (%) of CSII in children and adolescents	Uptake (%) of CSII in adults
Carlow Kilkenny	12.89% (10.30–15.44%)	39.36% (29.49–49.24%)	8.64% (6.33–10.95%)
Clare	4.93% (2.82–7.03%)	24.32% (10.50–38.15%)	3.01% (1.26–4.77%)
Cork Combined	7.60% (6.53–8.67%)	31.64% (26.14–37.13%)	4.43% (3.55–5.32%)
Donegal	9.59% (7.66–11.51%)	42.86% (34.66–51.05%)	3.49% (2.17–4.81%)
Dublin Combined	12.59% (11.72–14.36%)	38.68% (34.80–42.56%)	9.21% (8.39–10.03%)
Galway	10.69% (8.70–12.68%)	28.70% (20.43–36.96%)	7.95% (6.08–9.82%)
Kerry	4.31% (2.75–5.88%)	20.29% (10.80–29.78%)	2.43% (1.17–3.69%)
Kildare and Wicklow	15.03% (13.28–16.78%)	44.00% (37.85–50.15%)	9.63% (8.03–11.24%)
Laois and Offaly	10.72% (8.29–13.15%)	30.53% (21.27–39.79%)	7.03% (4.85–9.22%)
Limerick and Tipperary	7.75% (6.38–9.12%)	29.25% (23.12–35.37%)	4.03% (2.93–5.14%)
Longford Westmeath	9.67% (7.12–12.22%)	27.50% (17.72–37.28%)	6.56% (4.21–8.91%)
Louth	11.77% (9.33–14.21%)	38.78% (29.13–48.42%)	7.09% (4.97–9.21%)
Mayo	6.86% (4.70–9.02%)	12.64% (5.66–19.63%)	5.50% (3.36–7.65%)
Meath	15.76% (13.21–18.30%)	53.66% (44.85–62.47%)	8.79% (6.63–10.95%)
Roscommon	4.00% (1.44–6.56%)	18.52% (3.87–33.17%)	2.03% (0.06–4.00%)
Sligo, Leitrim, Cavan, Monaghan	8.55% (6.80–10.30%)	30.58% (22.37–39.79%)	5.32% (3.81–6.83%)
Waterford City and County	8.15% (5.73–10.57%)	20.45% (12.03–28.88%)	5.33% (3.11–7.55%)
Wexford	9.55% (7.42–11.68%)	36.00% (25.14–46.86%)	6.31% (4.42–8.20%)
**ALL IRELAND**	**10.51% (10.09–10.94%)**	**34.70% (32.86–36.53%)**	**6.81% (6.44–7.19%)**

Significant variation in the overall uptake of CSII between regions was observed from 4% (Co. Roscommon) to 15.8% in Co. Meath (*χ*^2^ = 213.6, *p <* 0.001; Table 2). High uptake was observed in areas close to the capital city – Dublin (12.6% in Dublin and its suburbs), in counties Kildare & Wicklow (15%) and Carlow & Kilkenny (12.9%). There were differences in regional uptake between the paediatric and adult populations. In the paediatric population the uptake varied significantly (*χ*^2^ = 105.2, *p <* 0.001; Fig. 1, Table 2) and was lowest in county Mayo (12.6%), and highest in county Meath (53.7%). The region with the lowest uptake in the paediatric population was still higher than any uptake levels in adults. In adults the uptake varied from 2% (county Roscommon) to 9.6% (counties Kildare and Wicklow) and the differences were significant (*χ*^2^ = 187.8, *p <* 0.001; Fig. 1, Table 2). See Table 2 for more detailed regional information.

## Discussion

This study found that 10.5% of those with type 1 diabetes were using CSII in Ireland in 2016. Overall, the uptake of CSII in children and adolescents was five-fold higher than in adults. The uptake was the highest, at 38%, in the youngest age-group (0–14 years) and was significantly lower with increasing age. There was significant geographical heterogeneity in CSII uptake with a four-fold variation in uptake across regions for children and adolescents, and five-fold for adults.

In comparison to other countries where CSII therapy is fully reimbursed (all Western European countries), the uptake in Ireland is relatively low. The average uptake in Nordic, Central and Western countries was 15–20% in 2010 [24]. The uptake in Ireland is even lower than in other developed countries with no public funding, where CSII is available mainly to those with private health insurance. In 2014, 12% of the population in Australia [25], and 59% of those with type 1 diabetes participating in the T1D Exchange clinic registry in the US [26] were using CSII. However, the T1D Exchange registry includes 16,061 participants from 76 diabetes US clinics (38 adult and 38 paediatrics), so this figure may not represent that national picture as a whole.

The uptake of CSII in children and adolescents in Ireland (34.7%) is similar to that in the UK where it was reported as 35.7% in those aged < 18 years in England and Wales in 2019 [27] and in the 2017 Scottish National Survey [15]. However, the uptake of CSII in these countries is generally lower than in other Western countries. The highest uptake of CSII in children and adolescents was observed in Slovenia (74%), with Sweden and Denmark also having > 50% uptake [28]. The study conducted within the SWEET network for paediatric diabetes centres (based on clinic databases from > 30 paediatric clinics) suggests an average of 44% uptake across Europe (2016) [28], and 60% of children and adolescents included in the US T1D Exchange clinic registry (38 clinics, 8483 participants aged < 18 years), [26] were using CSII in 2014.

In adults, the uptake of CSII in Ireland is also lower than in other countries where this mode of treatment is reimbursed [24]. Uptake has been reported to vary from 9.4% in Scotland [15], to 15% in England [29] and Italy [16] (data from 2017), 21% in Denmark (in one region) [30], 22% in Sweden (data from 2015) [10], 24% in Germany, Austria, Switzerland and Luxemburg (data from 2017) [31]. More recent data suggest that uptake of CSII in dutls from Germany only is 37% in 2017. [14]. Data from the US suggests that the uptake of CSII was even higher (59%) in adults in 2014 [26], but for reasons outlined above, unlike the German data, the US figure may not be representative of the population as a whole [14, 26]. Uptake of CSII in Irish adults is similar to Wales (6.7%) [29], and to findings of the national audit of CSII care in the UK conducted in 2012 [9]. The authors of the UK report concluded that this prevalence was “well below the expectations of the National Institute for Health and Clinical Excellence (NICE) guidelines (15-20%) or the European average (15%)” and, therefore, steps have been undertaken in the UK, in particular in England, to improve the uptake [15, 29, 32]. As a result, uptake in adults in England and Scotland has doubled since 2012 [15, 29], but this is still below that in the Nordic countries, Germany or the US [10, 26, 30, 31]. In Ireland, there are no standardized criteria or clinical recommendations for the use of CSII therapy in adults at present [33], which may be one of the factors for the low uptake of CSII. Commencement of CSII is usually at the discretion of the physician and team looking after the person with diabetes although aiming to optimize control, limit hypoglycaemia, improve hypoglycaemia awareness and personal preferences would be common indications. Having more firm guidelines could direct physicians to recommend CSII more often. On the other hand, bearing in mind lower uptake in the UK when comparing to Nordic countries, strict criteria, as those in the NICE guidelines, may have an impact on the poor uptake of CSII also. It is worth to note, that many health-care professionals in Ireland receive their postgraduate training (as well as training to provide CSII services) in the UK, therefore the NICE criteria are well-known by significant percentage of specialists in diabetes in Ireland. Other possible barriers to uptake, in particular in adults, might be similar to those explored by Italian researchers. According to the Third Italian Survey of CSII, high costs of CSII and lack of multidisciplinary teams (MDT) are perceived as limiting factors for CSII uptake [34]. The availability of CSII was explored more in-depth in a national survey of CSII services provision in adult clinics in Ireland. The scarcity of trained staff, as shown in the survey findings, means that full MDTs were not always available (e.g. lack of dietetic support), and the lack of MDT and perceived work overload were listed as barriers to CSII provision [35]. Another barrier might be related to people with diabetes lack of willingness to be attached to a device and a burden associated with technology use [36, 37].

The diversity in uptake of CSII between different age groups is common. Authors of the study conducted in Sweden concluded that people aged between 20 and 30 years were more than twice as likely to initiate use of CSII than those aged 40–50 years [38], and data from registries suggest that younger people with type 1 diabetes use CSII more often than older adults [15, 29]. CSII is recommended by ISPAD as a preferred mode of treatment in the youngest population [1]. CSII is often initiated in pre-school children due to their and their families’ needs related to unpredictable food patterns, low insulin requirements, reduction in the number of injections, ease of insulin delivery and needle-phobia [1], which helps explain why CSII is used more by younger people [29]. These needs have been recognized by the Irish National Paediatric Clinical Programme which introduced a model of care for the provision of CSII in children aged ≤5 years in 2012 [39]. This policy document recommends offering CSII to every child with type 1 diabetes under the age of 5 years, which may have contributed to the large difference in uptake between paediatric and adult populations.

Evidence on geographical variation in CSII uptake [15, 27, 29, 40] is lacking. Where evidence is available, for example, in Scotland, the variation was found to be two-fold from 27.1 to 60% in the paediatric population and 6.7 to 15.2% in adults [15] in 2017. This variation was not as high as that found in our study. There were four-fold differences between the overall uptake in Ireland and in children in adolescents, while a five-fold difference was observed in adults. Overall, the areas of high uptake seem to be centralized around Dublin both in the paediatric and adult populations. Low uptake seems to be more often in rural areas, such as Roscommon, Clare or Kerry. The rural-urban difference might partly explain such a heterogeneity, although there are exceptions such as Donegal which has one of the highest uptakes of CSII in children and adolescents despite being considered “rural”. Another explanation might be related to the age distribution of local populations. The youngest populations are in parts of Dublin and adjacent counties, whereas the oldest are in rural counties [41]. However some areas with older populations also have higher uptake than areas where the population is younger. This suggests that determinants are more complex than age, and rural-urban disparities. Previous data from Italy also shows geographical disparity even though the Italian health system covers the cost of devices; geographical disparity was explained by different regional regulations in terms of prescription rules and requirements [34]. In Ireland, prescription regulations are the same, but diversity in uptake could be affected by local health offices where decisions regarding funding are made [42].

This study aimed to estimate the uptake of CSII in Ireland and concludes that it is low both in the paediatric and adult populations. The reasons for such low uptake are not related to reimbursement since CSII is fully reimbursed in Ireland. The reasons are more complex, and may include lack of national standardized documents including guidelines for commencing CSII, the perceived excessive workload and the lack of resources [16, 34] This, however, has to be explored further with a use of more complex research methods.

The main strength of this study is that it is population-based, nationwide and based on objective data. Our regional findings relate to the residence of those with diabetes and not where they receive their diabetes care; thus the findings accurately describe local access to CSII. This is the first study describing the uptake of CSII in all regions and the entire population of people with type 1 diabetes in Ireland. Data based on prescriptions claimed for CSII sets are a reliable and accurate source of information regarding CSII utilization.

This study has some limitations. It was not possible to monitor discontinuation of CSII, where others have shown discontinuation rates in the range 1–4% [43]. Although the numbers using CSII reported are accurate, the uptake rates may be impacted on by the definition of type 1 diabetes cases, which was based on diabetes-specific prescriptions included in the pharmacy claims database. As information on the diagnosis (i.e. ICD-10 codes) is not available in the HSE-PCRS database and there is no diabetes registry in Ireland, some cases of people with type 2 diabetes receiving basal-bolus therapy or CSII may have been misclassified as type 1 diabetes. In addition, as the HSE-PCRS database is mainly used for the administrative purpose and does not contain any other medical information, there was no possibility to monitor the outcomes of CSII use, i.e. in levels of glycated haemoglobin (HbA_1c_). Moreover, because Continuous Glucose Monitoring (CGM) sensors are not included in the HSE-PCRS database (funding is covered from a different budget), we were unable to investigate the uptake of sensor-augmented insulin pump therapy use.

## Conclusions

Uptake of CSII remains low in Ireland when compared to other countries where, like Ireland, CSII is fully reimbursed. The uptake is five-fold higher in children/adolescents than in adults. Our regional findings accurately describe local access to CSII and suggest this access is unequal. This study highlights the potential under-utilisation of CSII in Ireland and suggests that further studies exploring potential barriers, both from the health-care providers’ and patients’ perspectives, are warranted. An understanding of the reasons for the low uptake of CSII will have important implications for improving the quality of care for people living with type 1 diabetes in Ireland. These findings will help to inform health service users and policymakers, and can help to support health-service planners in making decisions on health-service resource distribution. Also, our study suggests that in a country without a national diabetes register, routinely collected administrative pharmacy claims data can be utilized to estimate the uptake of CSII. Finally, this study adds to limited international evidence on the uptake of CSII therapy in those with type 1 diabetes.

## Supplementary information

**Additional file 1: Table S1.** The list of 18 geographical areas based on the matching between 32 HSE-PCRS Local Health Offices (LHOs) and 31 CSO County and City areas.

## Data Availability

The pharmacy claims data are managed by the HSE Primary Care Reimbursement Services and may be obtained in de-identified format for specific research purposes only. They are not permitted to be reused after analysis is completed. The formal permission to access to HSE-PCRS data granted for a limited period of use (6 months). To request access to the HSE-PCRS data please visit the https://www.hse.ie/eng/staff/pcrs/pcrs-publications/ and complete the Information Request form.

## Abbreviations

ATC classification: Anatomical Therapeutic Chemical classification
CGM: Continuous Glucose Monitoring
CI: Confidence intervals
CSII: Continuous Subcutaneous Insulin Infusion
CSO: Central Statistics Office
DP: Drug Payment Scheme
GMS: General Medical Scheme
HSE: Health Service Executive
HSE PCRS: Health Service Executive Primary Care Reimbursement Service
isCGM: intermittently scanned Continuous Glucose Monitoring
ISPAD: International Society for Pediatric and Adolescent Diabetes
LHO: Local Health Office
LTI: Long-Term Illness Scheme
MDI: Multiple Daily Injection
MDT: Multidisciplinary team
NICE: National Institute for Health and Care Excellence
PCRS: Primary Care Reimbursement Service
RCT: Randomized Controlled Trial
STROBE: Strengthening the reporting of observational studies in epidemiology
T1D Exchange: Type 1 Diabetes Exchange
UK: United Kingdom
US: United States
WHO: World Health Organization
