# Supplementary material for: Low uptake of continuous subcutaneous insulin infusion therapy in people with type 1 diabetes in Ireland: a retrospective cross-sectional study
Source: BMC Endocr Disord. 2020 Jun 23;20:92. doi: 10.1186/s12902-020-00573-w (PMC7310521; doi:10.1186/s12902-020-00573-w)
Supplement: Supplementary file 1 — Additional file 1: Table S1. The list of 18 geographical areas based on the matching between 32 HSE-PCRS Local Health Offices (LHOs) and 31 CSO County and City areas. [file 12902_2020_573_MOESM1_ESM.docx]

*Supplementary Material – Table S1*

The list of 18 geographical areas based on the matching between 32 HSE-PCRS Local Health Offices (LHOs) and 31 CSO County and City areas

| **32 Local Health Offices (PCRS data base)** | **31 CSO (Census) County and City areas** | **18 LHO-CSO geographical areas (matched and, combined)** |
| --- | --- | --- |
| South Dublin | South Dublin | Dublin Combined |
| Dublin South East | Dublin City |  |
| Dublin South City | Fingal |  |
| Dublin South West | Dun Laoghaire - Rathdown |  |
| Dublin West |  |  |
| Dublin North West |  |  |
| Dublin North Central |  |  |
| Dublin North |  |  |
| Kildare West Wicklow | Kildare | Kildare and Wicklow |
| Wicklow | Wicklow |  |
| Laois-Offaly | Laois | Laois and Offaly |
| Longford - Westmeath | Longford | Longford and Westmeath |
|  | Westmeath |  |
| Sligo | Sligo | Sligo, Leitrim (West Cavan), Cavan, Monaghan |
|  | Leitrim |  |
| Cavan-Monaghan | Cavan |  |
|  | Monaghan |  |
| Louth | Louth | Louth |
| Meath | Meath | Meath |
| Galway | Galway City | Galway |
|  | Galway County |  |
| Mayo | Mayo | Mayo |
| Roscommon | Roscommon | Roscommon |
| Donegal | Donegal | Donegal |
| Clare | Clare | Clare |
| Limerick |  | Limerick and Tipperary |
| North Tipperary - East Limerick | Limerick City and County |  |
| South Tipperary | Tipperary |  |
| Cork - South Lee | Cork City | Cork combined |
| Cork - North Lee | Cork County |  |
| West Cork |  |  |
| North Cork |  |  |
| Kerry | Kerry | Kerry |
| Carlow - Kilkenny | Carlow | Carlow and Kilkenny |
|  | Kilkenny |  |
| Waterford | Waterford City and County | Waterford |
| Wexford | Wexford | Wexford |
